# Evaluation of plasma antioxidant activity in women with early pregnancy losses

**DOI:** 10.7555/JBR.38.20240396

**Published:** 2025-05-27

**Authors:** Olga Gennadievna Tishkova, Lydmila Vasilievna Dikareva, Nadezhda Titovna Berberova, Maria Alexandrovna Polovinkina, Victoria Pavlovna Osipova

**Affiliations:** 1 Department of obstetrics and gynecology of the pediatric faculty with a postgraduate education course, Astrakhan State Medical University, Astrakhan, Astrakhan region 414000, Russia; 2 Department of chemistry, Astrakhan State Technical University, Astrakhan, Astrakhan region 414056, Russia; 3 Toxicology sector, Federal Research Centre Southern Scientific Centre of the Russian Academy of Sciences', Rostov-on-Don, Rostov region 344006, Russia

Dear Editor,

Early pregnancy loss is a condition whose relevance is determined not only by high incidence but also by the frequency of this pathology progressing into habitual miscarriage. According to the American Pregnancy Association, non-developing pregnancy (NDP), one of the forms of pregnancy loss, accounts for half of all miscarriages in the early stages^[1]^.

Oxidative processes in early pregnancy under physiological hypoxia play an important regulatory function in stimulating the expression of cytokines, heat shock proteins, growth and differentiation factors, and angiogenesis^[2]^. With the development and growth of the placenta, the need for oxygen increases sharply, as does the production of reactive oxygen species (ROS). However, under conditions of deficiency or depletion of the antioxidant defense system, ROS can damage virtually all molecular structures. As a result of the imbalance between the levels of ROS and antioxidants, oxidative stress develops in the body. In some cases, excessive placental oxidative stress in early pregnancy is the main cause of fetal death and developmental abnormalities^[3]^.

Currently, the evaluation of oxidative stress is carried out by measuring the levels of lipid and protein peroxidation products and by determining the blood levels of antioxidant activity (AOA) as an integral parameter that reflects the overall antioxidant status^[4]^. Previously, based on the obtained indicators of lipid and protein peroxidation, we demonstrated the development of oxidative stress in women with NDP^[5]^. However, there is currently no clear information regarding the plasma AOA levels in women with normal pregnancies and those with NDP. Therefore, the current study aimed to investigate the plasma activity of the antioxidant system during normal and non-viable pregnancies as a predictor of early pregnancy loss.

The current study was approved by the Institutional Review Board of Astrakhan State Medical University (extract from the minutes of meeting No. 3 of October 18, 2016) and was carried out following the standards and principles of the Declaration of Helsinki, with voluntary informed consent obtained from each participant. It was a prospective case-control study in which pairs were matched for age, weight, and social status. The study group consisted of 40 patients diagnosed with NDP at 5–9 weeks of gestation, and the control group included 40 women with normal pregnancies who decided to terminate their pregnancies at their own discretion during the same period. The exclusion criteria included patients with endocrine pathology and obesity, uterine factors of miscarriage, severe extragenital and infectious diseases, antiphospholipid syndrome, and congenital thrombophilia in the anamnesis, smokers, as well as women with confirmed pathological karyotypes of the fetus and women after the *in vitro* fertilization program. The plasma was obtained by drawing blood from a peripheral vein, followed by centrifugation at 1500 *g* for 10 min.

We used a complex of practical methods to detect AOA levels, because a large number of antioxidants make up the body's multi-stage antioxidant defense system, working through various mechanisms to inhibit oxidative processes and often exhibiting synergistic antioxidant effects^[6]^. Reagents were purchased from Sigma-Aldrich (St. Louis, MO, USA) and were used without further purification for the biological tests. The plasma АOА was determined using the stable 1,1-diphenyl-2-picrylhydrazyl radical (DPPH; Cat. #D9132), the cation radical of 2,2ʹ-azino-bis(3-ethylbenzothiazoline-6-sulfonic acid) diammonium salt (ABTS; Cat. #A1888), and the natural nitroxyl radical (NO) obtained from a solution of sodium nitroprusside (≥ 99%, CAS 13755-38-9)^[7]^. The cupric reducing antioxidant capacity (CUPRAC) assay was based on the ability of blood plasma components to reduce CuCl_2_ (Cat. #751944) in complex with neocuproine (Cat. #N1501)^[8]^. Superoxide dismutase (SOD) activity was measured by assessing the ability of the corresponding enzyme to inhibit the formation of the superoxide anion radical during the reaction of adrenaline (L-epinephrine) autooxidation in an alkaline medium (Cat. #E4250)^[9]^. Spectrophotometric measurements were carried out on a Multiskan Sky spectrophotometer (Thermo Fisher Scientific, Waltham, MA, USA) at the following wavelengths: 347 nm for SOD analysis, 450 nm for the CUPRAC test, 548 nm for the NO test, 517 nm for the DPPH test, and 734 nm for the ABTS test.

Statistical processing was performed using the Statistica 10 software package. Quantitative data with normal or approximately normal distribution were presented as the mean and standard deviation. For variables with a non-normal distribution, the data were presented as the median and interquartile range. The significance of differences between two independent samples was assessed using the parametric Student's *t*-test and the nonparametric Mann–Whitney test. The Holm-Bonferroni method was used to control the family-wise error rate. The relationship between quantitative variables was assessed using Spearman's rank correlation coefficient (*r*). Differences were considered statistically significant at *P* < 0.05.

The average age of the NDP and control groups was 30.5 and 30.9, respectively, with no significant difference between the two groups (confidence interval [CI]: 29.5–31.8, *P* = 0.8911). No statistical differences were found in the onset of menarche (*P* = 1.0000), regularity (*P* = 0.6712), and duration of the menstrual cycle (*P* = 0.8932) between the two groups. The analysis of reproductive outcomes showed that the number of term deliveries was significantly lower (*P* = 0.0016), while spontaneous miscarriages (*P* = 0.0003) and non-viable pregnancies (*P* = 0.0427) were significantly higher in the NDP group than in the control group. Diseases such as inflammation of the appendages (salpingo-oophoritis) in medical history (*P* = 0.0325), primary infertility (*P* = 0.0241), and benign ovarian tumors (*P* = 0.0245) were statistically more frequent in the NDP group than in the control group.

We conducted a preliminary correlation and regression analysis of some variables, including parental ages, unfavorable factors, bad habits, body mass index, *etc*. The calculated Pearson coefficients indicated that there was no significant difference in the results of the methods employed to determine AOA based on age and the presence of bad habits (*P* < 0.15). The most prominent negative correlation was found between body mass index and the results of SOD, ABTS, and DPPH tests (*P* < 0.05). Sample power analysis showed that the statistical power was 0.95 for DPPH, ABTS, and NO tests, 0.87 for the SOD assay, and 0.54 for the CUPRAC assay in the current study.

The analysis of plasma AOA levels in women of the NDP and normal groups is presented in ***Table 1***. After Bonferroni correction, only the *P*-value of the CUPRAC test was above the critical value of the adjusted significance level. For the other tests, statistically significant differences were observed. We observed a decrease in the inhibition of DPPH radical by 29.7%, and of ABTS, NO, and SOD by 20.1%, 6.5%, and 12.2%, respectively, in the NDP group compared with those in the normal group. The CUPRAC test did not reveal significant differences between the two groups (*P* = 0.0520).

**Table 1:** The plasma antioxidant activity levels in normal and NDP women

Methods	Control group (*n*=40)	NDP group (*n*=40)	*P*
DPPH (%)	52.8 (34.9, 68.2)	23.1 (15.1, 33.8)	0.0032
ABTS (%)	70.5 (49.6, 90.7)	50.4 (30.1, 61.3)	0.0037
CUPRAC (%)	10.3 (9.7, 10.4)	10.9 (10.4, 11.4)	0.0520
NO (%)	39.3 (35.8, 58.5)	32.8 (20.0, 35.6)	0.0021
SOD (%)	36.4 (21.1, 55.9)	24.2 (9.3, 38.9)	0.0153
The results of the experiment are presented as % inhibition relative to the control containing the same volume of water instead of the plasma sample. Data are presented as median and interquartile range. Abbreviations: NDP, non-developing pregnancy; DPPH, 1,1-diphenyl-2-picrylhydrazyl; ABTS, 2,2ʹ-azino-bis(3-ethylbenzothiazoline-6-sulfonic acid) diammonium salt; CUPRAC, cupric reducing antioxidant capacity; NO, nitroxyl; SOD, superoxide dismutase.			

Further, to assess the diagnostic significance, the sensitivity and specificity of all proposed tests were calculated. Among these, DPPH and ABTS tests demonstrated the greatest diagnostic value, with specificities of 62.5% (95% CI: 0.43–0.71) and 50% (95% CI: 0.31–0.64), respectively, and sensitivities of 57.7% (95% CI: 0.41–0.67) and 58.3 % (95% CI: 0.44–0.69), respectively (*P* < 0.05). Though the obtained sensitivity results are relatively low, they enable reliable identification of significant pathology while reducing errors in detecting abnormalities in healthy subjects.

**Figure 1:**
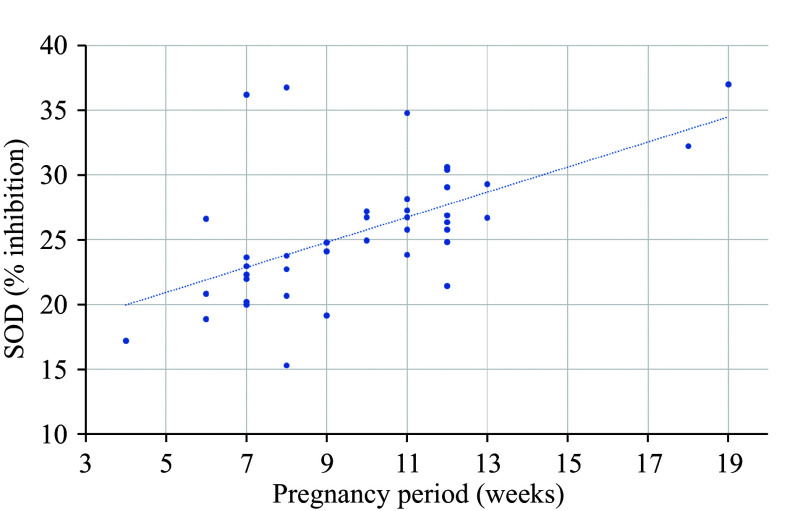
Dependence of superoxide dismutase (SOD) activity level on the gestational age at pregnancy termination. SOD activity level (% inhibition) of blood plasma from women with non-developing pregnancy (*n* = 40) depends on the gestational age at pregnancy termination.

In women with NDP, we found a negative correlation between the SOD activity level and the gestational age at pregnancy termination, with a correlation coefficient of −0.3657 (*P* < 0.05). Conversely, in women with normally developing pregnancies, a directly proportional relationship was observed between the SOD activity level and the gestational age, with a determination coefficient of *R*^2^ = 0.44, as shown in ***Fig. 1***. Additionally, a moderate positive correlation was established between the SOD activity level and the number of previous NDPs in medical history, with a correlation coefficient of 0.3816 (*P* < 0.05).

Thus, the current study demonstrated that in cases of NDP, there is a statistically significant decrease in antiradical and SOD activity, which confirms the role of oxidative stress in the pathogenesis of this pathology. The highest antiradical activity of blood plasma among the tested methods in the DPPH test is presumably explained by the hydrophobicity of the DPPH radical and may indicate a higher level of fat-soluble antioxidants (tocopherol, retinol, coenzyme Q10, vitamin D, *etc*.) than water-soluble antioxidants (ascorbic acid, B vitamins, flavonoids, *etc.*) in the plasma of early-stage pregnant women. Understanding the balance of antioxidants in the body is of great practical importance and will help avoid ineffective preventive supplementation during a treatment.

Most active antioxidants exhibit the properties of strong reducing agents. The reduction of radicals to the corresponding ions interrupts the chain reaction; therefore, the reducing potential reflects the ability of these compounds to regulate the redox status in plasma and tissues. The CUPRAC test allows evaluation of the ability of an antioxidant to reduce Cu(Ⅱ) ions to Cu(Ⅰ) ions. In the current study, the test did not show statistically significant differences between the two groups, which might be because Cu(Ⅱ) in both the free and bound states has a low reducing potential, and its interaction with the copper ions is more selective. However, the reduction of copper ions is an even more sensitive indicator of the prooxidant potential of reducing antioxidants. Thus, the obtained data indicate the need for further research.

Weak or absent correlations between the studied tests reflect the diversity of the mechanisms of the studied tests. The results of any of the studied tests may vary depending on the clinical situation, but other factors are unlikely to influence changes in the antiradical activity of the blood, since all analyses were carried out under identical conditions. Therefore, a combination of these tests can objectively enhance the assessment of patients' clinical conditions and may be useful for identifying the predominant mechanisms of the inhibitory effect of various antioxidants. Additionally, the studied tests have a number of advantages, including high reproducibility, ease of implementation, general availability of the necessary equipment, and sensitivity and selectivity with respect to antioxidants.

In conclusion, our study indicates the important role of oxidative stress in the pathogenesis of early pregnancy losses. A comprehensive assessment of plasma AOA levels can be a useful technique that allows not only prompt identification and prevention of pregnancy complications, but also accurate determination of a woman's reproductive health status at the pregravid preparation stage.

The work was carried out with the financial support of the Russian Science Foundation (Grant No. 23-13-00201).

Yours sincerely,Olga Gennadievna Tishkova^1,✉^, Lydmila Vasilievna Dikareva^1^, Nadezhda Titovna Berberova^2^, Maria Alexandrovna Polovinkina^3^, Victoria Pavlovna Osipova^3^ ^1^Department of Obstetrics and Gynecology of the Pediatric Faculty with a Postgraduate Education Course, Astrakhan State Medical University, Astrakhan, Astrakhan Region 414000, Russia;^2^Department of Chemistry, Astrakhan State Technical University, Astrakhan, Astrakhan Region 414056, Russia;^3^Toxicology Sector, Federal Research Centre Southern Scientific Centre of the Russian Academy of Sciences, Rostov-on-Don, Rostov Region 344006,Russia.^✉^Corresponding author: Olga Gennadievna Tishkova, E-mail: tishkov2003@mail.ru.
